# A Multivariate Generalizability Theory Approach to College Students' Evaluation of Teaching

**DOI:** 10.3389/fpsyg.2018.01065

**Published:** 2018-06-26

**Authors:** Guangming Li, Guiyun Hou, Xingjun Wang, Dong Yang, Hu Jian, Weijun Wang

**Affiliations:** ^1^Guangdong Key Laboratory of Mental Health and Cognitive Science, Center for Studies of Psychological Application, School of Psychology, South China Normal University, Guangzhou, China; ^2^School of Public Finance and Public Administration, Jiangxi University of Finance and Economics, Nanchang, China; ^3^Research Institute on Addictions, University at Buffalo, State University of New York, Buffalo, NY, United States

**Keywords:** multivariate generalizability theory, college teachers, teaching level evaluations, dependability index, college students

## Abstract

Teachers' teaching level evaluation is an important component in classroom teaching and professional promotion in the institutions of higher learning in China. Many self-made questionnaires are currently being administered to Chinese college students to evaluate teachers' classroom teaching performance. Quite often, due to the absence of strong educational, and psychological measurements and theoretical foundations for these questionnaires, their dependability remains open to doubt. Evaluation time points, the number of students, major type, and curriculum type were examined in relation to college students' perceptions on their teachers' classroom teaching performance, using Teachers' Teaching Level Evaluation Scale for Colleges (TTLES-C). Data were collected in a sample of 556 students at two time points from three Chinese universities and were analyzed using multivariate generalizability theory. Results showed that evaluations at the beginning of the spring semester produced better outcomes than did evaluations at the end of the fall semester, and 20 student evaluators were sufficient to ensure good dependability. Results also revealed that the evaluation dependability of science curriculum appeared higher than that of liberal arts curriculum. Recommendations were discussed on the evaluation criteria and mode.

## Introduction

College teachers' classroom teaching level is related to students' future success, particularly their professional development. Evaluations of teaching performance often have two primary purposes: administrative decision making and teaching improvement (McKeachie, 1997; Pike, 1998). In most cases, students are the only evaluators of the quality of teacher education, and their evaluations are assumed to have generated highly accurate and consistent outcomes (Hameed et al., 2015). Most universities exclusively use student evaluation of teaching (SET) instruments to provide information for the instructors to improve the quality of teaching. For example, in the U.S., institutions of higher education use some types of student evaluations of teaching instrument as a means of assessing instructors' instructional performance in courses (Dommeyer et al., 2002). Within this context, SETs affect both annual teaching performance and salary decisions as well as promotion and tenure decisions. Therefore, the validity and credibility of SETs data become important.

In order to effectively evaluate teachers' classroom teaching performance, a number of factors should be taken into account, such as the evaluation time, curricula, and raters' preference (e.g., Goe et al., 2008; Meyer et al., 2012; Gitomer et al., 2014). For most Chinese colleges, teaching evaluations are compulsory. Guided by this regulation, college students are all required to participate, and fill the self-made evaluation questionnaires regardless of the class size, although this tends to cost colleges a multitude of resources. Nevertheless, one defect embedded in these evaluations is the indiscrimination because questionnaires are administered to students without considering academic majors, learning habits, course goals, and individual preferences for the evaluated teacher(s). Quite often, in Chinese colleges the same lecturer delivers different courses to different classes throughout one semester (and even over an academic year), meaning that the lecturer receives a multitude of varied evaluation scores. This may bring about a problem when diverse evaluation results are integrated into one single benchmark. To be specific, for example, different students may have different preferences over different curricula—some “boring” curricula may demand more teaching skills, whereas some “interesting” curricula may not due to their close relationship with students' daily life or relevant teachers' individual charm in delivering these curricula. Similarly, students' expectations about their teachers may vary in majors and curricula. For example, teachers of liberal arts tend to focus on expounding theories and teachers of science majors may concentrate on the practical functions of theories, while teachers of engineering are inclined to attach more importance to the applications of some specific technologies. Accordingly, students' expectations about their teachers may fluctuate according to these teaching practices in different majors and curricula and in turn will impact their evaluations about their teachers.

Teaching evaluations are typically conducted at the end of fall semester, prior to the final exams (and students are the only raters) in Chinese colleges. We are worried about this, because in this case, teachers might retaliate against students by grading their final exams low if they failed to receive expected evaluation scores from students in these routine surveys. Obviously, college students are usually under the pressure of final exam grading. What is even worse, some students may care less about these evaluations than their teachers' grading of their final exams. Thus, students' evaluations of their teachers are most likely to be fraught with biases in this setting. So, evaluation time becomes a critical variable in evaluations of teaching (Wolfer and Johnson, 2003).

Several researchers have investigated the score reliability of SETs. However, their findings are mixed (Haskell, 1997). Some reported large reliability coefficients (Peterson and Kauchak, 1982; Seldin, 1984; Marsh and Bailey, 1993), but a few others (e.g., Simmons, 1996) reported inadequate score reliability coefficients. Most cited reasons for these mixed reliability coefficients included: (a) coefficient alpha's inability to identify multiple sources of measurement error; (b) each source of measurement error variance (e.g., raters, domain coverage, time when measurements are obtained) is separate and cumulative in its effect; (c) sources of measurement error can include interaction effects (Sun et al., 1997). In a nutshell, evaluations of teachers may be associated with a host of factors, such as time points, class size, majors, and curricula (Shin and Raudenbush, 2012; Casabianca et al., 2015). The failure to encompass these variables may deviate the evaluations from the objective, independent, and neutral ones, and thus endanger the reliability of the questionnaire. Thus, generalizability theory (GT) (Sun et al., 1997) has been advocated to assess the reliability of SET instead of using coefficient alpha, because the former addresses the multiple sources of measurement error typically found in student ratings.

In the classical test theory (CTT), the observed test score X is considered to be a linear model of the true score T and the error score E. The GT liberalizes the limitations of the CTT because GT differentiates the multiple sources of error that comprise E by using ANOVA-like procedures (Lord and Novick, 1968; Briggs and Wilson, 2007). In the GT terminology, the objective of a measurement is to measure the characteristics of the subject, and the facet is the potential source of measurement error excepted the measurement objective. For example, in an achievement test, the ability of the subject is the objective of a measurement, and the item, the rater, as well as the test form are the facets.

In general, the expected scores of the participants are different from the observed scores. The expected scores are obtained based on all possible facet conditions, but the observed scores are obtained based on the sampled facet conditions. The difference between the expected score and the observed score can be partially obtained based on the facet-based measurement error. The framework of GT mainly consists of two parts, one is G (generalizability) study and the other is D (decision) study. The purpose of G study is to separate the variance components into multiple error sources. The purpose of D study is to quantify the universe score variance, error variances, and measurement precision coefficients based on the G study (Brennan, 2001a). From this point of view, the estimation of variance components for a G study design is definitely of central concern.

In order to separate the error variance components, the generalizability theory adopts the concept of experimental design. According to the relationship between influencing factors, the design of GT is divided into cross design (×), nested design (:), and mixture design. We chose the simple single-facet measurement design (*p* × i) and used the generic terminology to illustrate the procedures of GT. In the *p* × i design, p represents the objective of a measurement and i represents the facet, and the *p* × i design means all persons need to answer all items. According to the fundamental assumption of GT, we believed that both the persons (*p*) and items (i) are sampled independently and randomly from the population of persons and items. We note *X*_*pi*_ as observed score of person p on item i. The grand mean across persons and items is defined as:

(1) $$\begin{array}{r} \mu =E_{p}E_{i}X_{pi}, \end{array}$$

the person-specific mean is noted as:

(2) $$\begin{array}{r} \mu_{p}=E_{i}X_{pi}, \end{array}$$

and the item-specific mean is noted as:

(3) $$\begin{array}{r} \mu_{i}=E_{p}X_{pi}. \end{array}$$

The linear model for *X*_*pi*_ can be written as:

(4) $$\begin{array}{r} X_{pi}=\mu \;\left(\text{grand mean}\right) \end{array}$$

$$\begin{array}{r} +\mu_{p}-\mu \;\left(\text{person effect}\right)\\ +\mu_{i}-\mu \;\left(\text{item effect}\right)\\ +X_{pi}-\mu_{p}-\mu_{i}+\mu \;\left(\text{residual}/\text{interaction effect}\right). \end{array}$$

The linear model for *X*_*pi*_ can also be written as:

(5) $$\begin{array}{r} X_{pi}=\mu +v_{p}+v_{i}+v_{pi,e}, \end{array}$$

where *v*_*p*_(person effect) = μ_*p*_−μ, *v*_*i*_(item effect) = μ_*i*_−μ, *v*_*pi, e*_(residual/interaction effect) = *X*_*pi*_−μ_*p*_−μ_*i*_+μ. ANOVA formulas and notation for G study *p* × *i* design are given in Table 1.

**Table 1:** ANOVA formulas and notation for G study *p* × i design.

**Effect (α)**	***df* (α)**	***T* (α)**	**MS (α)**	**$\hat{\sigma }$^2^(α)**
person (p)	n_p_-1	${\text{n}}_{\text{i}}\underset{p}{\sum }{\bar{X}}_{p}^{2}$	[T(p)–T(u)]/(n_p_-1)	[MS(p)-MS(pi)]/ *n*_*i*_
item (i)	n_i_-1	${\text{n}}_{\text{p}}\underset{i}{\sum }{\bar{X}}_{i}^{2}$	[T(i)–T(u)]/(n_i_-1)	[MS(i)-MS(pi)]/ *n*_*p*_
p × i, e	(n_p_-1) (n_i_-1)	$\underset{p}{\sum }\underset{i}{\sum }X_{pi\;}^{2}$	[T(pi)–T(p)–T(i)+ T(u)]/[(n_p_-1) (n_i_-1)]	MS(*pi, e*)
u	n_p_ × n_i_-1	n_p_${\text{n}}_{\text{i}}{\bar{X}}^{2}$		

In a subsequent D study, the decision to be made is based on the average. We take the *p* × *I* design for example, the observed score variance associated with the item facet is denoted $\frac{{\hat{\sigma }}^{2}(i)}{{n^{\prime }}_{i}}$, and the person by item interaction is denoted $\frac{{\hat{\sigma }}^{2}(pi,e)}{{n^{\prime }}_{i}}$. The measurement precision coefficients are denoted by generalizability coefficient (Eρ^2^) and dependability index (ϕ).

(6) $$\begin{array}{r} E\rho^{2}=\frac{{\hat{\sigma }}^{2}\left(p\right)}{{\hat{\sigma }}^{2}\left(p\right)+\frac{{\hat{\sigma }}^{2}\left(pi,e\right)}{n_{i}^{{}^{\prime }}}} \end{array}$$

(7) $$\begin{array}{r} \phi =\frac{{\hat{\sigma }}^{2}\left(p\right)}{{\hat{\sigma }}^{2}\left(p\right)+\frac{{\hat{\sigma }}^{2}\left(pi,e\right)}{n_{i}^{{}^{\prime }}}+\frac{{\hat{\sigma }}^{2}\left(i\right)}{n_{i}^{{}^{\prime }}}} \end{array}$$

Some studies have used GT to explore the factors that affect student evaluations of teaching. Shin and Raudenbush (2012) discussed the effects of class size, the number of evaluators, and the number of items on teaching evaluation results under the framework of GT. Some researchers have also used GT to explore the impact of evaluation time and evaluation methods on the evaluation results of teaching level (Casabianca et al., 2015). Huang et al. (1995) found that the graduate level students' curriculum assessment was more reliable using the generalizability analysis than undergraduate and intermediate levels. However, few studies have examined the application of GT in SET in Chinese college students. Given the current educational teaching systems, curriculum settings, and professional settings in China, it is necessary to use GT to study the influencing factors of SET in Chinese universities.

GT enables researchers to build contexts between the objects of measurement and the facets of measurement based on different conditions and defined factors (Saunders et al., 1989; Marcoulides and Goldstein, 1990; Saunders, 1992; Hill et al., 2012). When the overall estimate (i.e., the generalizability coefficient or the dependability index) is high, the obtained scores from a measurement scale can be generalized across the given facets (Spooren et al., 2014). GT meets the demand for teachers' teaching level evaluations and can generate more reliable results. In the present study, we demonstrated the use of GT in analyzing the teachers' teaching level data in a sample of Chinese college students. The generalization coefficient applies to the norm-referenced test, and the dependability index applies to the standard-referenced test. In this study, we used the dependability index as the measurement precision coefficients. Specifically, this study examined whether variables such as evaluation time points, the number of students in class, types of major and curriculum would affect Chinese college students' evaluations of teaching performance of their teachers.

## Materials and methods

### Participants

The sample included 566 students (54.6% females) aged 17–21 from 19 classes (6 classes for freshmen, 7 for sophomores, and 6 for juniors) in three universes. Data were collected in the 2014–2015 academic year at two time points (i.e., Time 1, at the end of the first semester, fall semester; Time 2, at the beginning of the second semester, spring semester). 98.6% (*n* = 558, 55.4% females) completed the survey questionnaire at T2. Seven classes focused on liberal arts curriculums and the other 12 classes involved science curriculums (Table 2). With respect to major types of the sample, 4 classes provided engineering courses, 10 classes provided science courses, and the other 5 classes focused on liberal arts majors. The sample evaluated 19 teachers and their teaching performance, and the same curriculum was taught throughout the whole academic year in each class based on curriculum types and major types.

**Table 2:** Student numbers, major type, and curriculum type for each class.

**Class**	**1**	**2**	**3**	**4**	**5**	**6**	**7**	**8**	**9**	**10**	**11**	**12**	**13**	**14**	**15**	**16**	**17**	**18**	**19**
N^1^	21	34	68	36	36	64	28	22	26	25	25	17	22	30	26	23	21	22	20
N^2^	19	31	60	29	35	74	21	26	25	28	27	23	22	27	26	20	23	23	19
Major	E	A	A	A	S	S	E	E	S	S	E	A	S	S	S	S	S	S	A
Curriculum	S	A	A	A	S	S	S	S	A	A	S	A	S	S	S	S	S	S	A

*N^1^ = Student numbers in each class at the end of the first semester (T1). N^2^ = Student numbers in each class at the beginning of the second semester (T2). A, Liberal arts; S, Science; E, Engineering*.

### Measures

We developed the Teachers' Teaching Level Evaluation Scale for Colleges (TTLES-C) to evaluate Chinese college teachers' teaching level. TTLES-C included 25 questions on a 5-point scale (from 1 = *Disagree at all* to 5 = *Agree very much*). We performed a series of Confirmatory Factor Analyses (CFA; Geiser, 2012) to identify the dimensions of the scales using a pilot sample data (*n* = 524) collected in 2013, prior to our formal research. Results indicated 5 dimensions (and each of these dimensions was examined using 5 items and averaged): teaching methods (e.g., “the teacher is good at using multi-media, such as lantern slide, models, films, for teaching.”), teaching content (e.g., “the teacher introduces us the present trend of the subject and the background of the learning content.”), teaching attitudes (e.g., “the teacher prepares for each class very well.”), teaching organizations (e.g., “the teacher encourages us to contact with him/her after class by phone or e-mails.”), and teaching effects (e.g., “through the study, I grasp the basic principles and theories of the curriculum.”). The model fit was acceptable (Hu and Bentler, 1999; Schermelleh-Engel et al., 2003): χ^2^/*df* = 3.283, CFI/TLI = 0.927/0.918, RMSEA = 0.066, SRMR = 0.039. Internal consistency for the overall scale (i.e., all 25 items included) was α = 0.96 (T1) and α = 0.96 (T2). Internal consistency for the subscales was: teaching methods T1 α = 0.87, T2 α = 0.87; teaching content T1 α = 0.80, T2 α = 0.82; teaching attitudes T1 α = 0.90, T2 α = 0.88; teaching organizations T1 α = 0.85, T2 α = 0.83; and teaching effects T1 α = 0.91, T2 α = 0.89. The correlations among these dimensions were 0.68 ≤ *r* ≤ 0.78 (T1) and 0.59 ≤ *r* ≤ 0.79 (T2). Li and Zhang (2017) used TTLES-C and examined student evaluations of teaching in a sample of 543 Chinese college students and reported the same patterns of internal consistency reliabilities. Demographic information was also assessed, including grade, gender, major, curriculum, and gender of the teacher.

### Procedures

The Academic Affairs Offices of the 3 study colleges provided a list of “teachers who only give one course in one semester.” Nineteen classes were randomly selected. In total, we investigated 19 teachers (and 19 courses) and their teaching performance. All the 19 courses were mandatory, and had the same workload (two 45-min lessons a week). Data were collected during the class in 45 min using a paper/pencil version survey administered to all students in these classes, first at the end of the first semester (T1, before the final exam; fall semester) and then at the beginning of the second semester (T2, spring semester). Research staff were trained before they administered the survey. Student assent was obtained, and this study received approval documents from the targeted university's research ethics board (Institutional Review Board).

### Analytic plan

Data were collected and saved with .txt format, and analyzed in mGENOVA software. Following the recommendations of Brennan (2001a,b) see also (Shavelson and Webb, 1991), we used multivariate GT modeling to produce the results from the G study and the D study. We first analyzed data from T1 and T2 separately, and found that data collected in spring (i.e., T2) had higher dependability index. We then conducted the following analyses using the data collected at T2 (i.e., data collected in spring).

In a multivariate GT model, each content area represents one fixed category (v), and a series of items or questions (i) are included and allowed to be correlated with each category. The present study used a two facets nested design, students and items for a facet, with students nested teacher and crossed items. We considered several main factors, including evaluation time points, the number of students, students' majors, and curriculum types.

In the sense of a univariate GT, our data format could be referred to as (s:t) × (i:h), where s, t, i, and h represent student, teacher, item, and content facets, respectively. But due to the fixed content facet, a multivariate generalizability study design was generated (s^∙^:t^∙^) × i°, with the number of levels for the fixed facet as n_h_. The solid circle, ^∙^, represents that the student and teacher facets are crossed with the fixed multivariate variable (i.e., content), while the empty circle, °, designates that the item facet is nested within the fixed multivariate variable. As a result, there are two random effects within each of the five fixed content categories and any single item is only associated with one single content category. The following are the mathematical equations for each of our content categories (Wu and Tzou, 2015):

(8) $$\begin{array}{r} X_{\text{stih}1}=\mu_{h1}+\upsilon_{th1}+\upsilon_{ih1}+\upsilon_{s:th1}+\upsilon_{tih1}+\upsilon_{is:th1},\\ {\text{X}}_{\text{stih}2}=\mu_{h2}+\upsilon_{th2}+\upsilon_{ih2}+\upsilon_{s:th2}+\upsilon_{tih2}+\upsilon_{is:th2},\\ X_{\text{stih}3}=\mu_{h3}+\upsilon_{th3}+\upsilon_{ih3}+\upsilon_{s:th3}+\upsilon_{tih3}+\upsilon_{is:th3},\\ {\text{X}}_{\text{stih}4}=\mu_{h4}+\upsilon_{th4}+\upsilon_{ih4}+\upsilon_{s:th4}+\upsilon_{tih4}+\upsilon_{is:th4},\\ X_{\text{stih}5}=\mu_{h5}+\upsilon_{th5}+\upsilon_{ih5}+\upsilon_{s:th5}+\upsilon_{tih5}+\upsilon_{is:th5}. \end{array}$$

In these equations, μ denotes the grand mean. *X*_*sti*_ is an observed score, representing one student evaluates one teacher on a certain item. ν_t_ represents a teacher's effect and ν_i_ represents an item's effect. ν_s:t_ represents the effect of students nested teacher. ν_ti_ represents the interaction effect between teacher and item. ν_is:t_ represents the three-way interaction effect among students nested teacher, teacher and item or non-system effect which has not been measured. The total variation from the observed scores is equal to these effects, i.e., $\sigma_{\left(\text{Xstih1}\right)}^{2}=$ $\sigma_{\left(\text{th1}\right)}^{2}+$ $\sigma_{\left(\text{ih1}\right)}^{2}+$ $\sigma_{\left(\text{s:th1}\right)}^{2}+$ $\sigma_{\left(\text{tih1}\right)}^{2}+$ $\sigma_{\left(\text{is:th1}\right)}^{2}$. For each fixed content, $\sigma_{\left(\text{t}\right)}^{2}$ is the teacher variance component which represents the differences between the teachers' teaching levels. $\sigma_{\left(\text{i}\right)}^{2}$ is the item variance component which refers to the differences between items. $\sigma_{\left(\text{s:t}\right)}^{2}$ is the variance component of students nested teachers which stems from different students. $\sigma_{\left(\text{ti}\right)}^{2}$ is the variance component of teacher and item which refers to the differences between teachers in different items. $\sigma_{\left(\text{is:t}\right)}^{2}$ is the variance component of the three-way interaction effect among teacher, student, and students nested teacher, non-interactive system error source, and others unknown.

## Results

The minimums, maximums, means, standard deviations, and correlations for the five dimensions of our measures at T1 and T2 are presented in Table 3.

**Table 3:** Minimums, maximums, means, standard deviations, and correlations for five dimensions.

	**Descriptive statistics**						**Correlations**				
	**Variable**	***N***	**Min**	**Max**	**Mean**	***SD***	**1**	**2**	**3**	**4**	**5**
Time 1	1. Teaching methods	566	6.00	25.00	20.08	3.87	–				
	2. Teaching content	566	6.00	25.00	19.53	3.65	0.78^**^	–			
	3. Teaching attitudes	566	5.00	25.00	20.58	4.09	0.76^**^	0.71^**^	–		
	4. Teaching organizations	566	5.00	25.00	18.80	4.12	0.68^**^	0.69^**^	0.68^**^	–	
	5. Teaching effects	566	5.00	25.00	19.11	4.18	0.69^**^	0.71^**^	0.71^**^	0.76^**^	–
Time 2	1. Teaching methods	558	6.00	25.00	20.60	3.63	–				
	2. Teaching content	558	6.00	25.00	19.81	3.60	0.76^**^	–			
	3. Teaching attitudes	558	5.00	25.00	21.00	3.57	0.79^**^	0.70^**^	–		
	4. Teaching organizations	558	5.00	25.00	19.41	3.64	0.68^**^	0.75^**^	0.66^**^	–	
	5. Teaching effects	558	5.00	25.00	19.29	3.81	0.63^**^	0.67^**^	0.59^**^	0.74^**^	–

*1. Teaching methods; 2. Teaching content; 3. Teaching attitudes; 4. Teaching organizations; 5. Teaching effects, similarly hereinafter*.

** *Correlation is significant at the 0.01 level (2-tailed)*.

### Effects of evaluation time points on evaluation results

#### G study

The estimated G study variance and covariance components for the (s^∙^:t^∙^) × i° design are presented in Table 4 for the two time points data (i.e., T1, the end of the first semester, as can be seen in the upper part of the table; T2, the beginning of the second semester, see the lower part). Results showed that teaching attitudes had highest variance component among the five indicators both at T1 (0.14583; at the end of the first semester, before the final exam, fall semester) and at T2 (0.14239; at the beginning of the second term, spring semester), indicating that students most likely rated their teachers' teaching attitudes to be the most important indicator in their evaluations of teaching quality, regardless of the evaluation time points.

**Table 4:** Variance and covariate components from G study, effect of evaluation time points.

**Variables**	**Teaching methods**	**Teaching content**	**Teaching attitudes**	**Teaching organizations**	**Teaching effects**
**T1, AT THE END OF THE FIRST SEMESTER**					
*t*	**0.13019**				
	0.10469	**0.09399**			
	0.14004	0.11330	**0.14583**		
	0.12967	0.10746	0.13908	**0.13731**	
	0.11058	0.10174	0.12105	0.11664	**0.10511**
**T2, AT THE BEGINNING OF THE SECOND SEMESTER**					
*t*	**0.12136**				
	0.09890	**0.09116**			
	0.13333	0.10405	**0.14239**		
	0.11660	0.10404	0.12474	**0.12074**	
	0.09298	0.10002	0.10042	0.11618	**0.12024**

*The bold values are variance components*.

#### D study

**Figure 1:**
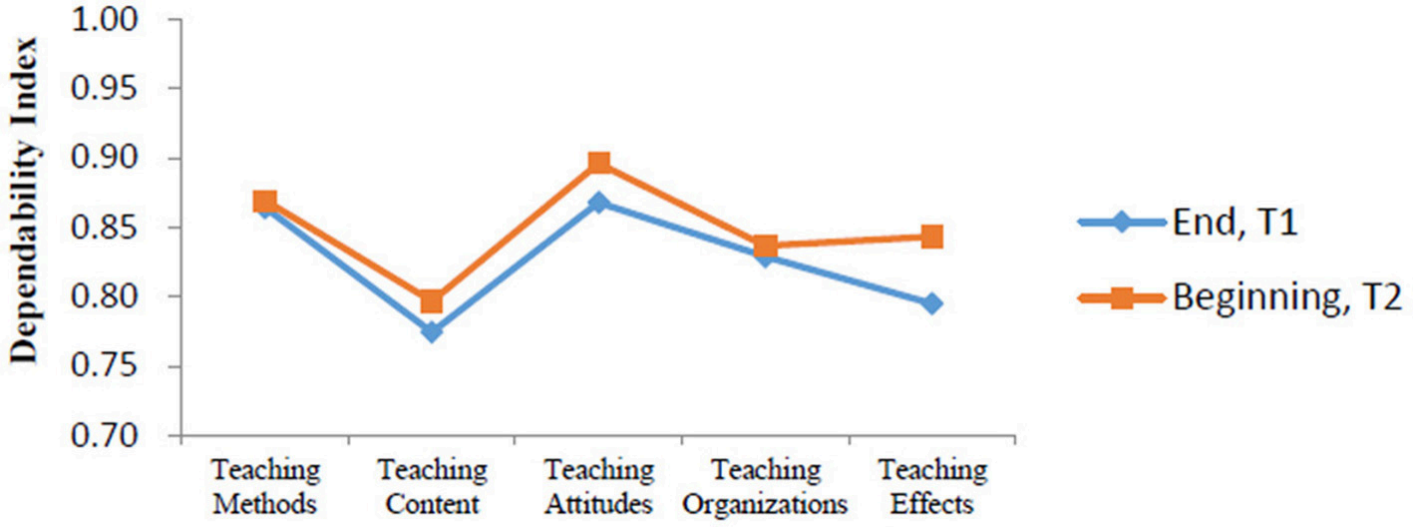
Dependability indexes for two different evaluation time points.

We compared the dependability indexes for the evaluations at two time points. Results indicated that sample sizes of D study for all facets within each content area in this part were similar to those found in the G study. Our D study produced good comprehensive dependability indexes at T1 (0.88513) and at T2 (0.89951). Five dependability indexes for T1 and T2 are showed in Figure 1. Consequently, our findings suggested that it was more reliable to have teachers' teaching level evaluated at the beginning of the second term (T2) rather than at the end of the first term (T1, before the final exams), according to the dependability indexes for each content area and the comprehensive dependability indexes for the two evaluation time points.

### Effects of student numbers on evaluation results

#### G study

Table 5 presents the G study variance and covariance components for the (s^∙^:t^∙^) × i° design. Among the five dimensions, teaching attitudes had highest variance component (0.14239). Students perceived teaching attitudes as the most important among these dimensions.

**Table 5:** Variance components and covariance components from g study, effect of student numbers.

**Variables**	**Teaching methods**	**Teaching content**	**Teaching attitudes**	**Teaching organizations**	**Teaching effects**
*t*	**0.12136**				
	0.09890	**0.09116**			
	0.13333	0.10405	**0.14239**		
	0.11660	0.10404	0.12474	**0.12074**	
	0.09298	0.10002	0.10042	0.11618	**0.12024**

*The bold values are variance components*.

#### D study

The design for the D study was (S^∙^:t^∙^) × I°, and the symbolic meaning was the same as in the G study. In this design, the capital letter (S, I) represented the mean value for the sample group. The D study sample sizes for all facets within each content area in this part were the same as those found in the G study. As showed in Table 6, the dependability indexes were between 0.79677 and 0.89603, statistically indicating that our scale was a reliable tool.

**Table 6:** Dependability index from D study.

	**Teaching methods**	**Teaching content**	**Teaching attitudes**	**Teaching organizations**	**Teaching effects**
Phi	0.86912	0.79677	0.89603	0.83643	0.84312

*Phi, Dependability index*.

**Figure 2:**
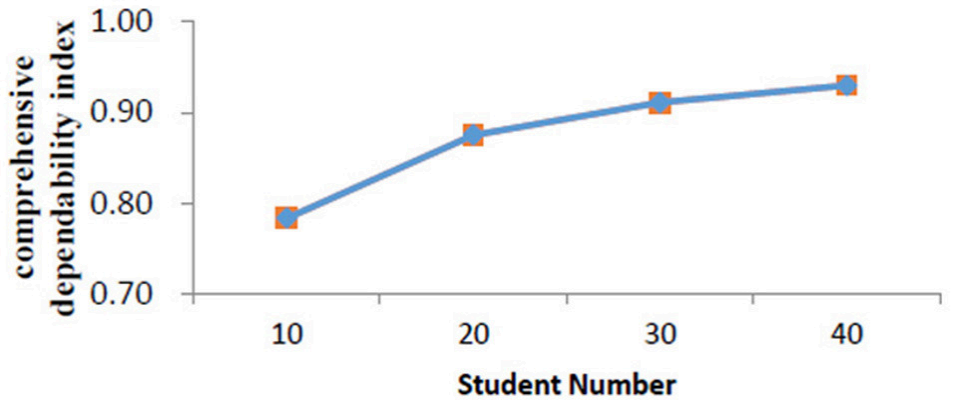
Comprehensive dependability index for different student numbers.

We then performed a series of D study by fixing the number of students to 10, 20, 30, and 40. Figure 2 presents relationships between student numbers and related comprehensive dependability index. Results showed that comprehensive dependability indexes elevated with the increases of the student number. For example, when the student number was fixed to 10, the comprehensive dependability index was 0.78. When the number was up to 20, the comprehensive dependability index reached as high as 0.88. However, we did not find a significantly increased dependability when the sample size was over 20.

### Effects of major type on evaluation results

#### G study

In order to examine the influence of major types on the evaluation results, we analyzed three subgroups of classes, which were picked from three categories of classes including four engineering classes (*n* = 93), four randomly selected liberal art classes (*n* = 102), and 4 science classes (*n* = 94). Table 7 presents the estimated G study variance and covariance components for the (s^∙^:t^∙^) × i° design for these three major types. Liberal arts students and engineering students perceived teaching attitudes to be more important than other indicators (the variance components for teaching attitudes were 0.08156 and 0.50233 for liberal arts students and engineering students, respectively), whereas science students reported teaching effects as the most important (0.13145) in the teaching quality evaluations.

**Table 7:** Variance components and covariance components from G study, effect of major type.

**Variables**	**Teaching methods**	**Teaching content**	**Teaching attitudes**	**Teaching organizations**	**Teaching effects**
**LIBERAL ARTS STUDENTS**					
*t*	**0.07947**				
	0.05078	**0.02326**			
	0.08676	0.05153	**0.08156**		
	0.05060	0.02694	0.05013	**0.02578**	
	0.03516	0.01902	0.03688	0.02922	**0.02120**
**SCIENCE STUDENTS**					
*t*	**0.06168**				
	0.04066	**0.07482**			
	0.06431	0.03933	**0.05363**		
	0.05129	0.07716	0.04720	**0.05575**	
	0.03491	0.11325	0.03393	0.09036	0.**13145**
**ENGINEERING STUDENTS**					
*t*	**0.42079**				
	0.29187	**0.22119**			
	0.46202	0.32770	**0.50233**		
	0.37893	0.26412	0.41724	**0.32636**	
	0.33216	0.26183	0.37925	0.30879	**0.31431**

*The bold values are variance components*.

#### D study

**Figure 3:**
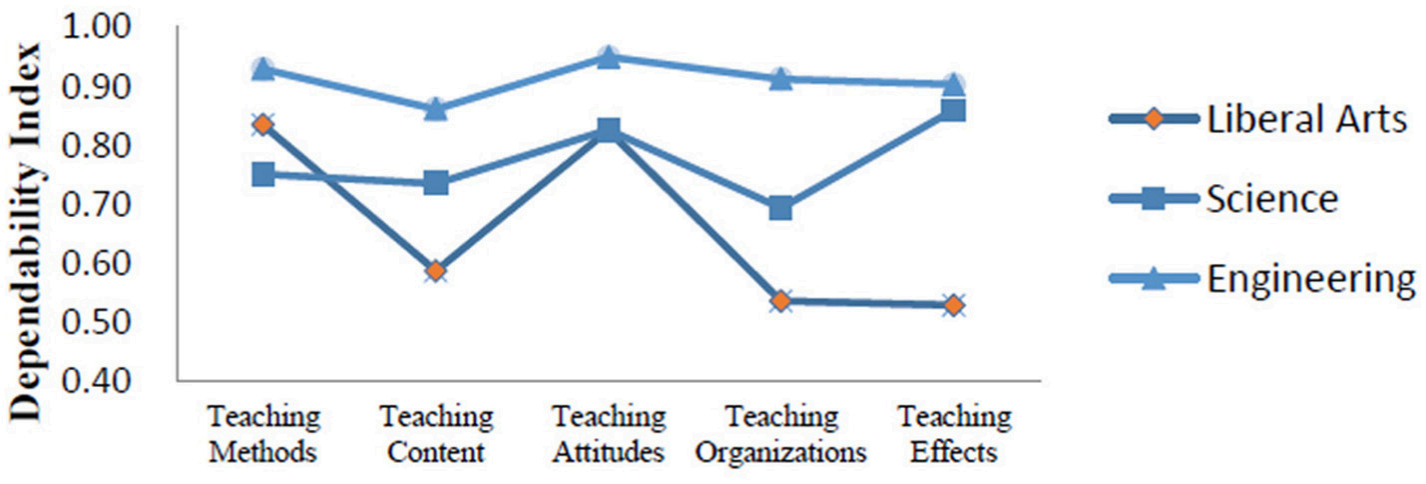
Dependability indexes for three major types.

Figure 3 shows the dependability indexes produced from the D study in three major types of students. The D study sample sizes for all facets within each content area in this part were the same as those found in the G study. According to the comprehensive dependability indexes, evaluations for engineering students were highly consistent (0.94), followed by science students (0.87) and liberal arts students (0.79).

### Effects of curriculum type on evaluation results

#### G study

Our G study involved the seven liberal arts classes (*n* = 215) and seven randomly selected science classes (*n* = 232). Table 8 summarizes the estimated G study variance and covariance components for the (s^∙^:t^∙^) × i° design for two types of curriculum. Students of liberal arts curriculum reported higher scores in teaching attitudes (the variance component, 0.04885) than in other indicators, whereas students of science curriculum reported higher scores in teaching effects (0.11177) than other indicators. These findings revealed that when evaluating their teachers' teaching quality, students from liberal arts curriculum rated teaching attitudes as the most important content area, while students from science curriculum rated teaching effects as the most important.

**Table 8:** Variance components and covariance components from G study, effect of curriculum type.

**Variables**	**Teaching methods**	**Teaching content**	**Teaching attitudes**	**Teaching organizations**	**Teaching effects**
**LIBERAL ARTS CURRICULUM**					
*t*	**0.03142**				
	0.01864	**0.00473**			
	0.03947	0.01774	**0.04885**		
	0.01927	0.00852	0.02472	**0.00976**	
	0.01449	0.00578	0.02040	0.01244	**0.00595**
**SCIENCE CURRICULUM**					
*t*	**0.07196**				
	0.06673	**0.07007**			
	0.07574	0.06784	**0.07235**		
	0.08180	0.08495	0.08671	**0.10843**	
	0.04794	0.07585	0.05336	0.09645	**0.11177**

*The bold values are variance components*.

#### D study

**Figure 4:**
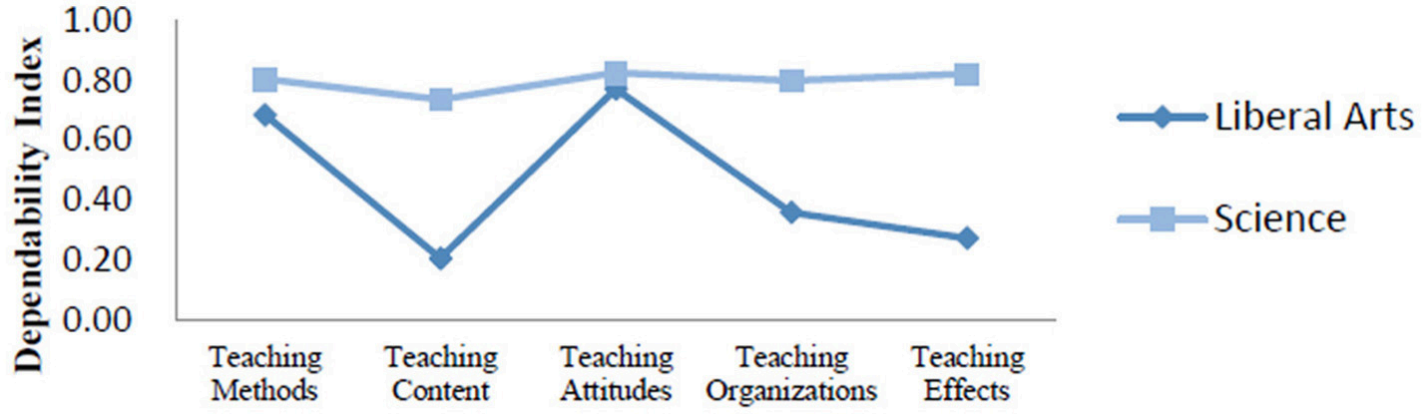
Dependability indexes for different curricula.

We compared the dependability indexes for the two types of curriculum (Figure 4). Findings indicated that D study sample sizes for all facets within each content area in this part were the same as those found in the G study. The ensuing analysis produced a comprehensive dependability index of 0.87 for science curriculum and 0.65 for liberal arts curriculum.

As showed in Figure 4, the dependability indexes of science curriculum for five evaluation indicators were around 0.80, indicating that students' evaluations were relatively consistent. However, the dependability indexes of liberal arts curriculum were low and these evaluations were somewhat not consistent. Specifically, for the liberal arts curriculum, the dependability indexes of teaching content, teaching organizations, and teaching effects were < 0.40. It was possible that liberal arts students had difficulty judging the performance of their teachers in terms of these three content areas. In addition, the comprehensive dependability index for the liberal arts curriculum was as low as 0.65, suggesting that our measurement scale needs to be improved to evaluate teaching quality with respect to the liberal arts curriculum.

## Discussion and conclusion

Teachers' classroom teaching level evaluation is compulsory in Chinese institutions of higher learning. In most cases, teachers designed their own questionnaires and they often lacked reliable dependability. Despite this, teachers still used students' rates on these surveys to improve their teaching level, for example, through changing teaching strategies and methods. University personnel and administrators also used these results as a basis for teachers' promotion and tenure decisions. In this study, we used multivariate GT to explore the effects of evaluation time points, the number of students in class, types of major, and curriculum on evaluation results of teaching performance using a sample of Chinese college students. Generalizability theory enabled us to divide the total error into student's error, item's errors, teacher's errors, their interaction errors, and residual error. Our G studies showed that the variance components and interactions varied among evaluation content areas. There were two large variance components for different facets: the interaction of the highest level and the student facet (s: t). The interaction of the highest level may have been confounded with some uncontrolled sources of variance (and errors) about which we know little yet, and this should be taken into consideration in future research. The large variance components for student facet (s: t) may suggest that students understood the evaluation dimensions (e.g., content areas) differently, which in turn may have fluctuated their ratings on college teachers' teaching performance.

### Effect of evaluation time points

Although college students' understanding and perceptions of their teachers' performance may change with the advancement of the curricula in an academic year, our study showed that students perceived their classroom teachers' teaching attitudes to be the most important indicator in their teaching performance at both evaluation time points (i.e., at the end of the first semester, fall semester, and at the beginning of the second semester, spring semester). We also found that rated scores received at the beginning of the spring semester were more reliable than those obtained at the end of the fall semester (before the final exams). It is possible that Chinese college students may feel more comfortable to rate their teachers' teaching level (and express their opinions) and take these evaluations more seriously at the beginning of the spring semester. If they have to fill the evaluation questionnaires at the end of the fall semester (before the final exams), they may be concerned about their exams and worry that these evaluations will impact their final exam scores (e.g., teachers who have received poor evaluation scores from students may retaliate against students with poor grades), and so they may not evaluate their teachers' teaching level faithfully. Thus, the evaluations of teaching in the first semester could be carried out at the beginning of the spring semester to ensure that the evaluation results are more reliable.

Some researchers suggested that the evaluation time should depend upon the purpose of evaluations of teaching—teaching improvement or administrative decision making (McKeachie, 1997; Oermann et al., 2018). Wolfer and Johnson (2003) argued that, for the purpose of teaching improvement, the evaluation of teaching should be conducted during the semester or even at the beginning of the semester, because teaching improvement needs sufficient time, and evaluating at the end of the semester cannot help teachers to make adjustments in time. However, students also need time to get to know their teachers to make correct assessments. For the purpose of administrative decision making (e.g., faculty assessment, promotion and tenure decisions), the evaluations of teaching should focus on the dependability. From this perspective, Wolfer and Johnson (2003) argued that the evaluation of teaching should be conducted at the end of the semester. Some scholars also noted that the evaluation of teaching at the time of the exam approaching had an impact on the evaluation score (Abbott et al., 1990) and should not be arranged on the day of the end of the course (Frey, 1976).

Our study using two time points suggests that evaluation time is an important factor that influences the SET, and evaluations of teaching performance should be carried out at the beginning of the second semester over an academic year (note that one course is taught to the same class in both semesters by the same teacher in Chinese universities), when we assume that students have been familiar with their teachers, and this evaluation time will also avoid the time of final exams, regardless of the purposes of these evaluative assessments.

### Effect of student numbers

According to the current scenario of Chinese college teachers' daily tasks, each teacher is allocated one or several courses at a fixed classroom during a fixed time period (e.g., the whole academic year). Generalizability theory (rather than the CTT) holds that a single evaluation with high level can well detect one's teaching level, which can save much time, money, and labor for relevant colleges. Our findings indicated that the dependability index of an evaluation for one teacher was acceptable when more than 10 students were involved in the analysis, as this would produce a dependability index of 0.78. Although more raters would lead to a better dependability, we did not find significant increases in dependability when the sample size was over 20, which was consistent with previous studies (e.g., Chang and Hocevar, 2000). Specifically, to ensure a dependability index of 0.80 or more, 20 students were needed in the evaluation.

These were important findings. In our daily practices in Chinese universities, the academic department (and even higher-level university administrative offices) sometimes use one or two students' comments on teachers' teaching, regardless of whether these opinions are positive or negative. The current study showed that one or two students' assessment of teachers' teaching was unreliable. The evaluation of a too small class (e.g., 5–6 students) often produces high variances of rating systems (Chang and Hocevar, 2000; Kane and Staiger, 2002). On the other hand, when the number of students exceeds 20, the student number variable will have relatively little influence on the dependability. This means that, in terms of dependability, when the number of students exceeds a certain value, the impact of the student number will be less obvious. It is unknown whether small classes tend to provide more positive evaluations than large classes (Chang and Hocevar, 2000; Wolfer and Johnson, 2003; Oermann et al., 2018). Future studies should consider the class size as a condition in assessing teachers' teaching quality rated by students.

### Effect of major type

This study also examined effects of majors among liberal arts, science, and engineering students. We found that liberal arts students and engineering students paid more attention to their teachers' professional attitudes, but science students rated teaching effects as the most important indicator in their teachers' performance. These findings suggest that students' major type be considered when college teachers design their teaching plans. We also found that dependability indexes of engineering majors for the five evaluation indicators were higher than those for science majors and liberal arts majors. Specifically, teaching attitudes had highest dependability index for engineering majors and liberal arts majors, but for science majors, teaching effects had highest dependability index. However, the lowest dependability index for science majors was teaching attitudes, and the outcome for liberal arts majors and engineering majors was teaching effects and teaching content respectively. Meanwhile, the comprehensive dependability indexes across these three majors demonstrated that the evaluation results from engineering were the most reliable, followed by science and liberal arts. These findings suggest that, in order to reduce the influence of major type on the evaluation results or to increase the comprehensive dependability indexes, researchers should adjust the weight for each evaluation indicator considering the major type. For example, when liberal arts students are asked to evaluate their teachers, researchers can elevate the weight for teaching attitudes but lower the weight for teaching effects at the same time.

### Effect of curriculum type

Our findings showed that students of liberal arts curriculum viewed teaching attitudes as the most important indicator, whereas students of science curriculum perceived teaching effects as the most important. Accordingly, we suggest that liberal arts teachers should place enough emphasis on their teaching attitudes while science teachers should place special attention and focus on teaching effects in their daily teaching. We also found that dependability indexes of science curriculum across all evaluation indicators were high and consistent, but those of liberal arts curriculum fluctuated and appeared inconsistent. It is possible that science teachers may have a tedious way of teaching, while liberal arts teachers may have a more personal charm (due to their curriculum features). Students did not like a tedious teaching style and their evaluations were consistent with their preference. In the meantime, some students may like teachers who often cite from the classics or ancient works when their teachers introduce background knowledge, some others may like teachers who have a passion in their teaching, and their evaluation rates may vary, which may lead to a low dependability index. Consequently, colleges should clarify the evaluation criteria before getting students to evaluate their teachers.

It is notable that students' attitudes toward curricula may affect their evaluations on teachers (Wolfer and Johnson, 2003; Oermann et al., 2018). If students are interested in a curriculum itself, they may feel more enthusiastic about attending classes, and thus may rate their teachers more positively. On the contrary, if they show little interest to a curriculum, they may feel bored with specific class teaching and choose to play truants, and thus they may rate their teachers low in the evaluation. We also believe that comprehension deviations from different curricula may arise when students read the scale. Liberal arts curriculum is tied to people's daily life and lays emphasis on the ability of speaking skills (e.g., liberal arts curriculum requires deep understanding of subject knowledge and students pay more attention to cultural understanding about theories), students may tend to rate liberal arts lecturers high in their evaluation when rating on these items. However, since science curriculum highlights the function of its applicability and values controlling experimental data (e.g., science curriculum requires skilled use of knowledge, and science students focus more on practical use), science teachers might not take these factors (e.g., speaking skills) seriously, and consequently they may receive relatively low scores on these items in the evaluations. Therefore, in order to eliminate the influence of course types, teachers should be separately rated according to their curriculum types.

In summary, the results of the present study indicated that the evaluation time points, the number of students in class, types of major and curriculum were associated with the SET. Reliabilities of SET can be increased through the following aspects: (1) evaluations of classroom teaching performance should be carried out at the beginning of the second semester over an academic year; (2) to ensure a dependability index of 0.80 or higher, 20 students are needed in the evaluation; (3) in order to reduce the influence of the major type on the evaluation results or to increase the comprehensive dependability indexes, researchers should adjust the weight for each evaluation indicator considering the major type; and (4) in order to eliminate (or limit) the influence of course types, teachers should be separately rated according to their curriculum types.

## Limitations

Although multivariate models in the present study enabled us to analyze a number of factors at the same time, we included only four key factors (i.e., evaluation time, the number of students, students' majors, and curriculum types). Other variables were not considered, such as gender, grade, teachers' physical appearance (Oghazi, 2015; Wolbing and Riordan, 2016), teaching experience (Maulana et al., 2015), and pedagogic environment (Pleschová and McAlpine, 2016). Only 19 teachers were assessed, and they taught one curriculum for both semesters over an academic year. In practice, many Chinese college teachers are required to teach more than one course. This context should be taken into account in future study. In a most recent study in a sample of Canadian college students, Vaillancourt (2013) found that students rated their professors low in reaction to receiving poor grades in students' evaluation of teaching. Although our study showed that evaluations conducted at the beginning of the spring semester produced better evaluation results, we did not know if this was somewhat also the case in our Chinese student sample (e.g., whether students rated their teachers low in reaction to receiving poor grades in the final exams last semester). In addition, although our data were longitudinal, most of our analyses only focused on T2 data.

The TTLES-C was a newly developed scale to assess classroom teaching quality. Although we conducted a series of CFA (Geiser, 2012) using an independent sample as a pilot study before we conducted the present study, also Li and Zhang (2017) provided evidence on the internal consistency reliabilities of the total scale (averaged 25 items) and of the five subscales, we were short of external validity evidence for the scale. This means that the findings in this study should be interpreted with caution. Our future study needs to address this issue.

## Author contributions

All authors contributed to the design of the study, analysis or interpretation of the data. All authors gave the final approval to the current version of the manuscript.

### Conflict of interest statement

The authors declare that the research was conducted in the absence of any commercial or financial relationships that could be construed as a potential conflict of interest. The reviewer LP and handling Editor declared their shared affiliation.

## References

[B1] Abbott R. D. Wulff D. H. Nyquist J. D. Ropp V. A. Hess C. W. (1990). Satisfaction with processes of collecting student opinions about instruction: the student perspective. J. Educ. Psychol. 82, 201–206. 10.1037/0022-0663.82.2.201

[B2] Brennan R. L. (2001a). Generalizability Theory. New York, NY: Springer-Verlag.

[B3] Brennan R. L. (2001b). Manual for mGENOVA. IA: Iowa Testing Programs. Iowa City, IA: University of Iowa.

[B4] Briggs D. C. Wilson M. (2007). Generalizability in item response modeling. J. Edu. Meas. 44, 131–155. 10.1111/j.1745-3984.2007.00031.x

[B5] Casabianca J. M. Lockwood J. R. McCaffrey D. F. (2015). Trends in classroom observation scores. Educ. Psychol. Meas. 75, 311–337. 10.1177/0013164414539163 29795823 PMC5965595

[B6] Chang L. Hocevar D. (2000). Models of generalizability theory in analyzing existing faculty evaluation data. Appl. Meas. Edu. 13, 255–275. 10.1207/S15324818AME1303_3

[B7] Dommeyer C. J. Baum P. Chapman K. S. Hanna R. W. (2002). Attitudes of business faculty towards two methods of collecting teaching evaluations: paper vs. online. Assess. Eval. High. Educ. 27, 455–462. 10.1080/0260293022000009320

[B8] Frey P. W. (1976). Validity of student instruction ratings: does timing matter? J. High. Educ. 47, 327–336.

[B9] Geiser C. (2012). Data Analysis With Mplus. New York, NY: Guilford Press.

[B10] Gitomer D. H. Bell C. A. Qi Y. McCaffrey D. F. Hamre B. K. Pianta R. C. (2014). The instructional challenge in improving teaching quality: Lessons from a classroom observation protocol. Teach. Coll. Rec. 116, 1–32.

[B11] Goe L. Bell C. A. Little O. (2008). Approaches to Evaluating Teacher Effectiveness: A Research Synthesis. Washington, DC: National Comprehensive Center for Teacher Quality.

[B12] Hameed F. Ali A. Hameed A. Saleem Z. Javed Y. (2015). Teacher evaluation: the role of gender. Qual. Quant. 49, 1779–1789. 10.1007/s11135-014-0054-3

[B13] Haskell R. E. (1997). Academic freedom, tenure, and student evaluation of faculty: galloping polls in the 21st century. Educ. Policy Anal. Arch. 5:10 10.14507/epaa.v5n6.1997

[B14] Hill H. C. Charalambous C. Y. Kraft M. A. (2012). When rater reliability is not enough: teaching observation systems and a case for the generalizability study. Educ. Res. 41, 56–64. 10.3102/0013189X12437203

[B15] Hu L. T. Bentler P. M. (1999). Cutoff criteria for fit indexes in covariance structure analysis: conventional criteria versus new alternatives. Struct. Equ. Model. 6, 1–55. 10.1080/10705519909540118

[B16] Huang C. Guo S. Druva-Roush C. Moore J. (1995). A Generalizability Theory Approach to Examining Teaching Evaluation Instruments Completed by Students. (ERIC Document Reproduction Service No. ED 394984). Columbus, OH: Clearinghouse on Adult, Career, and Vocational Education.

[B17] Kane T. J. Staiger D. O. (2002). The promise and pitfalls of using imprecise school accountability measures. J. Econ. Perspect. 16, 91–114. 10.1257/089533002320950993

[B18] Li G. M. Zhang M. Q. (2017). Weight effect analysis of multivariate generalizability theory for teaching level evaluation of college teachers. Psychol. Dev. Educ. 33, 122–128.

[B19] Lord F. M. Novick M. R. (1968). Statistical Theories of Mental Test Scores. Reading, MA: Addison-Wesley.

[B20] Marcoulides G. A. Goldstein Z. (1990). The optimization of generalizability studies with resource constraints. Educ. Psychol. Meas. 50, 761–768. 10.1177/0013164490504004

[B21] Marsh H. W. Bailey M. (1993). Multidimensional students' evaluations of teaching effectiveness: a profile analysis. J. High. Educ. 64, 1–18. 10.1080/00221546.1993.11778406

[B22] Maulana R. Helms-Lorenz M. Grift W. V. D. (2015). A longitudinal study of induction on the acceleration of growth in teaching quality of beginning teachers through the eyes of their students. Am. Biol. Teach. 51, 225–245. 10.1016/j.tate.2015.07.003

[B23] Greenwald A. G. (1997). Student ratings: the validity of use. Am. Psychol. 52, 1218–1225. 10.1037/0003-066X.52.11.1218 9357332

[B24] Meyer J. P. Cash A. H. Mashburn A. (2012). Occasions and the reliability of classroom observations: alternative conceptualizations and methods of analysis. Educ. Assess. 16, 227–243. 10.1080/10627197.2011.638884

[B25] Oermann M. H. Conklin J. L. Rushton S. Bush M. A. (2018). Student evaluations of teaching (set): guidelines for their use. Nurs. Forum. 10.1111/nuf.12249 [Epub ahead of print]. 29345731

[B26] Oghazi P. (2015). Beautiful teaching and good performance. J. Bus. Res. 69, 1887–1891. 10.1016/j.jbusres.2015.10.074

[B27] Peterson K. Kauchak D. P. (1982). Teacher Evaluation: Perspectives, Practices, and Promises. Salt Lake City, UT: University of Utah, Graduate School of Education.

[B28] Pike C. K. (1998). A validation study of an instrument designed to measure teaching effectiveness. J. Soc. Work Educ. 34, 261–271. 10.1080/10437797.1998.10778922

[B29] Pleschová G. McAlpine L. (2016). Helping teachers to focus on learning and reflect on their teaching: what role does teaching context play? Stud. Educ. Eval. 48, 1–9. 10.1016/j.stueduc.2015.10.002

[B30] Saunders P. F. (1992). Alternative solutions for optimization problems in generalizability theory. Psychometrika 57, 351–356. 10.1007/BF02295423

[B31] Saunders P. F. Theunissen T. J. Baas S. M. (1989). Minimizing the number of observations: a generalization of the Spearman-Brown formula. Psychometrika 54, 587–589. 10.1007/BF02296398

[B32] Schermelleh-Engel K. Moosbrugger H. Müller H. (2003). Evaluating the fit of structural equation models: Tests of significance and descriptive goodness-of-fit measures. Methods Psychol. Res. Online 8, 23–74.

[B33] Seldin P. (1984). Changing Practices in Faculty Evaluation. San Francisco, CA: Jossey-Bass.

[B34] Shavelson R. J. Webb N. M. (1991). Generalizability Theory: A Primer, Vol. 1. Thousand Oaks, CA: Sage Publications.

[B35] Shin Y. Raudenbush S. W. (2012). Confidence bounds and power for the reliability of observational measures on the quality of a social setting. Psychometrika 77, 543–560. 10.1007/s11336-012-9266-4 27519780

[B36] Simmons T. L. (1996). Student evaluation of teachers: professional practice or punitive policy. Shiken: JALT Testing and Evaluation SIG Newsletter 1, 12–19. Retrieved from: http://hosted.jalt.org/test/sim_1.htm

[B37] Spooren P. Mortelmans D. Christiaens W. (2014). Assessing the validity and reliability of a quick scan for student's evaluation of teaching. Results from confirmatory factor analysis and G Theory. Stud. Educ. Eval. 43, 88–94. 10.1016/j.stueduc.2014.03.001

[B38] Sun A. Valiga M. J. Gao X. ACT (1997). Using generalizability theory to assess the reliability of student ratings of academic advising. J. Exp. Educ. 65, 367–379. 10.1080/00220973.1997.10806611

[B39] Wolfer T. A Johnson M. M (2003). Re-evaluating student evaluation of teaching. J. Soc. Work Educ, 39, 111–121. 10.1080/10437797.2003.10779122

[B40] Vaillancourt T. (2013). Students aggress against professors in reaction to receiving poor grades: an effect moderated by student narcissism and self-esteem. Aggress. Behav. 39, 71–84. 10.1002/ab.21450 22997048

[B41] Wolbing T. Riordan P. (2016). How beauty works. Theoretical mechanisms and two empirical applications on students' evaluation of teaching. Soc. Sci. Res. 57, 253–272. 10.1016/j.ssresearch.2015.12.009 26973043

[B42] Wu Y. F. Tzou H. (2015). A multivariate generalizability theory approach to standard setting. Appl. Psychol. Meas. 39, 507–524. 10.1177/0146621615577972 29881023 PMC5978517

